# Hematopoietic cells emerging from hemogenic endothelium exhibit lineage-specific oxidative stress responses

**DOI:** 10.1016/j.jbc.2024.107815

**Published:** 2024-09-24

**Authors:** Harmke Biezeman, Martina Nubiè, Leal Oburoglu

**Affiliations:** 1 Molecular Medicine and Gene Therapy, Lund Stem Cell Center, Lund University, Lund, Sweden; 2 Division of Gene and Cell Therapy, Institute for Regenerative Medicine, University of Zurich, Zurich, Switzerland

**Keywords:** induced pluripotent stem cells, endothelial to hematopoietic transition, hematopoiesis, erythropoiesis, Nrf2, hypoxia, glutathione, oxidative stress

## Abstract

During human embryogenesis, distinct waves of hematopoiesis give rise to various blood cell types, originating from hemogenic endothelial (HE) cells. As HE cells reside in hypoxic conditions in the embryo, we investigated the role of hypoxia in human endothelial to hematopoietic transition and subsequent hematopoiesis. Using single-cell RNA sequencing, we describe hypoxia-related transcriptional changes in different HE-derived blood lineages, which reveal that erythroid cells are particularly susceptible to oxidative stress, due to decreased NRF2 activity in hypoxia. In contrast, nonerythroid CD45^+^ cells exhibit increased proliferative rates in hypoxic conditions and enhanced resilience to oxidative stress. We find that even in normoxia, erythroid cells present a clear predisposition to oxidative stress, with low glutathione levels and high lipid peroxidation, in contrast to CD45^+^ cells. Intriguingly, reactive oxygen species are produced at different sites in GPA^+^ and CD45^+^ cells, revealing differences in oxidative phosphorylation and the use of canonical *versus* noncanonical tricarboxylic acid cycle in these lineages. Our findings elucidate how hypoxia and oxidative stress distinctly affect HE-derived hematopoietic lineages, uncovering critical transcriptional and metabolic pathways that influence blood cell development.

At different developmental stages, transient and successive hematopoietic systems arise in the human embryo through waves and provide the embryo with blood cells. The first primitive wave occurs in the blood islands of the yolk sac (YS) and gives rise to megakaryocytes, macrophages, and primitive erythrocytes (1). A second, definitive wave in the YS results in more mature multipotential hematopoietic progenitor cells called erythro-myeloid progenitors (2, 3) and lymphoid progenitors (4, 5). The final wave is also a definitive wave through which hematopoietic stem cells (HSCs) emerge from the aorta–gonad–mesonephros region, and migrate to the fetal liver where they will expand and mature (6, 7). These HSCs are capable of differentiating into all types of blood cells (8, 9) and will ultimately reside in the bone marrow after birth. The HSCs, erythro-myeloid progenitors, and primitive erythrocytes that arise during these hematopoietic waves have been demonstrated to come from a subset of endothelial cells with hemogenic potential, named hemogenic endothelial (HE) cells (10, 11, 12). Through endothelial to hematopoietic transition (EHT), HE cells change from adherent and spindle-shaped to detached and round cells, and start expressing hematopoietic markers (13).

Human induced pluripotent stem cells (hiPSCs) have provided the possibility of studying blood formation by *in vitro* generation of HE cells (14). Using this model, we have previously shown that both glycolysis and oxidative phosphorylation (OXPHOS) increase during EHT, and the emergence of different hematopoietic cell types depend on distinct metabolic pathways (15, 16). Cellular metabolism is critically influenced by microenvironmental factors such as oxygen levels and oxidative stress that may arise from the latter (17). Although cells derived from HE reside in hypoxia *in vivo* (18), research on the role of hypoxia in human EHT and subsequent hematopoiesis is limited. Interestingly, the oxygen sensor hypoxia-inducible factor-1A (HIF-1α) was found to be crucial in the transition from HE to HSCs in both mice and zebrafish (18, 19). Specifically, it was shown that glucose metabolism controls HSC formation in zebrafish through reactive oxygen species (ROS) production and *hif1a* modulation (19). However, it is still unclear how specific HE-derived hematopoietic populations are affected by hypoxic conditions and how these populations counteract oxidative stress.

In this study, we sought to understand how low oxygen levels affect hematopoietic populations deriving from HE. Using HE cells derived from hematopoietic differentiation of human induced pluripotent cells (iPSCs), we investigated the role of oxidative stress, glutathione metabolism, and canonical/noncanonical tricarboxylic acid (TCA) cycle pathways in specifying HE-derived erythroid and nonerythroid populations. Here, we uncover striking differences in how oxidative stress affects and is dealt with by different hematopoietic populations following their emergence from HE.

## Results

### Single-cell analysis of HE-derived populations reveals transcriptional changes in different blood lineages in hypoxia

**Figure 1:**
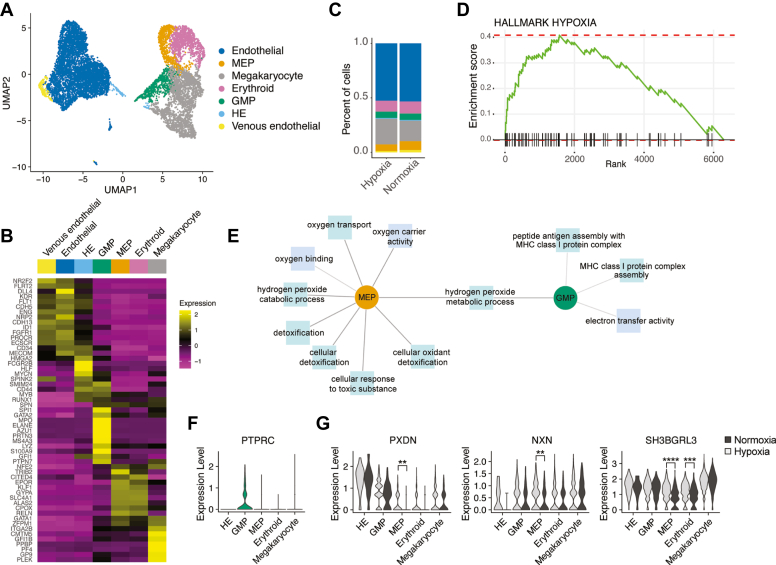
**scRNAseq reveals a hypoxia-induced cellular response to oxidative stress in MEPs.** iPSC-derived HE cells were cultured in hypoxia (4% O_2_) or normoxia (ambient) for 72 h and analyzed by scRNAseq. *A*, UMAP visualization of scRNAseq data with combined samples and colored by endothelial and hematopoietic clusters. *B*, heatmap showing average expression levels of cell type-specific markers. *C*, bar graph showing proportions of each cluster in hypoxia and normoxia conditions. *D*, enrichment score for the Hallmark hypoxia pathway (top upregulated pathway) obtained following gene set enrichment analysis of hypoxia *versus* normoxia conditions. *E*, cytoscape representation of Toppcluster analysis of the top significant DEGs in hypoxia *versus* normoxia in MEP and GMP clusters. *F*, Violin plot of *PTPRC* expression in hematopoietic clusters. *G*, Violin plots show expression of DEGs in antioxidant response pathways depicted in (*E*) in all hematopoietic clusters. DEG, differentially expressed gene; GMP, granulocyte-monocyte progenitor; HE, hemogenic endothelial; iPSC, induced pluripotent stem cell; MEP, megakaryocyte-erythrocyte progenitor; scRNAseq, single-cell RNA sequencing.

To gather comprehensive data on the effects of hypoxic conditions on the EHT process and the hematopoietic populations formed thereafter, we performed a multiome analysis with combined single-cell RNAseq and ATAC-seq on HE-derived populations following 72 h of subculture at 4% O_2_ (termed hypoxia for simplicity) or ambient oxygen levels (normoxia). First, we analyzed our single-cell RNAseq dataset and discerned seven clusters: Venous endothelial, endothelial, HE, granulocyte-monocyte progenitor (GMP), megakaryocyte-erythrocyte progenitor (MEP), megakaryocyte (megakaryocyte-biased progenitors) and erythroid (erythroid-biased progenitors) populations (Fig. 1*A* and cell numbers per cluster and per condition are indicated in Table 1).

**Table 1:** Cell numbers per cluster

Cluster	Hypoxia	Normoxia	Total
Venous endothelial	112	36	148
Endothelial	4531	875	5406
HE	68	13	81
GMP	500	96	596
MEP	516	132	648
Erythroid	856	181	1037
Megakaryocyte	2024	308	2332
Total	8607	1641	10,248

Cell identities were inferred using at least ten population-specific markers for each cell type (Fig. 1*B*), based on previous findings (20, 21, 22, 23, 24). Namely, venous endothelial cells expressed higher levels of vein-specific *NR2F2* and *FLRT2* genes, and low level of the arterial-specific *DLL4* gene. Both endothelial populations expressed well-described endothelium-specific genes such as *KDR*, *FLT1*, *CDH5*, *ENG*, and *ID1*. These genes were also expressed in HE cells at lower levels, and characteristically, HE cells also expressed HE-specific *FCGR2B* (CD32) and early hematopoietic markers (*CD34*, *MECOM*, *HMGA2*, *HLF*, *MYCN*, *SPINK2*, *CD44*, *MYB*, and *RUNX1*) but not CD43 (*SPN*), which is induced at a later stage during EHT (25). The GMP population expressed myeloid *SPI1*, neutrophil-specific *MPO*, *ELANE*, *AZU1*, *PRTN3*, *LYZ*, and monocyte-specific S100A9 genes. The MEP cells expressed specific markers *GATA1* and *ZFPM1*, as well as shared erythroid markers with the erythroid cluster such as *KLF1*, *GYPA*, *SLC4A1*, and *ALAS2*. Based on the high level of shared genes with the erythroid cluster, the MEP population we detect here appears to have a strong bias toward the erythroid lineage. Lastly, megakaryocyte cluster cells expressed *ITGA2B*, *PPBP*, *PF4*, *GP9*, and *PLEK* (Fig. 1*B*).

Next, we assessed the proportions of each cell type in hypoxia and normoxia conditions and observed comparable fractions for each population (Fig. 1*C* and Table 2), suggesting that oxygen levels may not affect the initial emergence of different hematopoietic lineages. We performed a gene set enrichment analysis (26) on differentially expressed genes (DEGs) in hypoxia as compared to normoxia and found that the top ten upregulated pathways included the hypoxia signature (Fig. 1*D*), as well as Wnt/β-catenin and transforming growth factor-β signaling pathways (Fig. S1*A*) which have been previously shown to be associated with hypoxia (27, 28). On the other hand, the top ten downregulated pathways in hypoxia included the ROS pathway, the oxidative phosphorylation signature, and MYC targets (Fig. S1*A*), in line with the previous findings (29, 30, 31). Moreover, we performed a transcription factor-transcriptional targets analysis *via* DoRothEA (32) and found that the HIF1A regulon was enhanced in hypoxia *versus* normoxia conditions, for all clusters (Fig. S1*B*). Intriguingly, we observed higher levels of *HIF1A* transcripts in nonerythroid CD45^+^ cells compared to GPA^+^ erythroid cells (Fig. S1*C*).

**Table 2:** Cell proportions per cluster

Cluster	Hypoxia	Normoxia	Average
Venous endothelial	0.013012664	0.021937843	0.01747525
Endothelial	0.526431974	0.533211456	0.52982172
HE	0.007900546	0.007921999	0.00791127
GMP	0.058092250	0.058500914	0.05829658
MEP	0.059951203	0.080438757	0.07019498
Erythroid	0.099453933	0.110298598	0.10487627
Megakaryocyte	0.235157430	0.187690433	0.21142393
Total	1	1	

To understand whether hypoxia leads to transcriptional changes in the earliest emerging hematopoietic cells, MEP and GMP, we assessed the top significant DEGs in hypoxia as compared to normoxia using ToppCluster (33), with a cutoff above 0.25-fold difference and an adjusted *p*-value of less than 0.05 (Wilcoxon Rank Sum test). As expected for MEP, we found hits such as oxygen carrier activity, oxygen transport, and oxygen binding, which are crucial processes for erythropoiesis (Fig. 1*E*). For the GMP cluster but not for MEP, we detected enrichment of MHC class I protein complex assembly. In line with this, MHC-I was previously shown to arise simultaneously with CD45 (nonerythroid pan-hematopoietic marker) in maturing HSCs (34). Indeed, we found that CD45 (*PTPRC*) was specifically expressed in the GMP cluster (Fig. 1*F*).

Intriguingly, we detected processes such as cellular oxidant detoxification and cellular response to toxic substance for the MEP cluster but not for the GMP cluster (Fig. 1*E*). The DEGs involved in antioxidant response included peroxidasin (*PXDN*), nucleoredoxin (*NXN*), and a redoxin-related gene (*SH3BGRL3*) and were significantly upregulated in the MEP cluster in hypoxia (Fig. 1*G*). These results led us to hypothesize that the MEP population (and resulting erythroid cells) may be more sensitive to oxidative stress that may result from hypoxic conditions.

### HE-derived erythroid cells are particularly susceptible to oxidative stress

**Figure 2:**
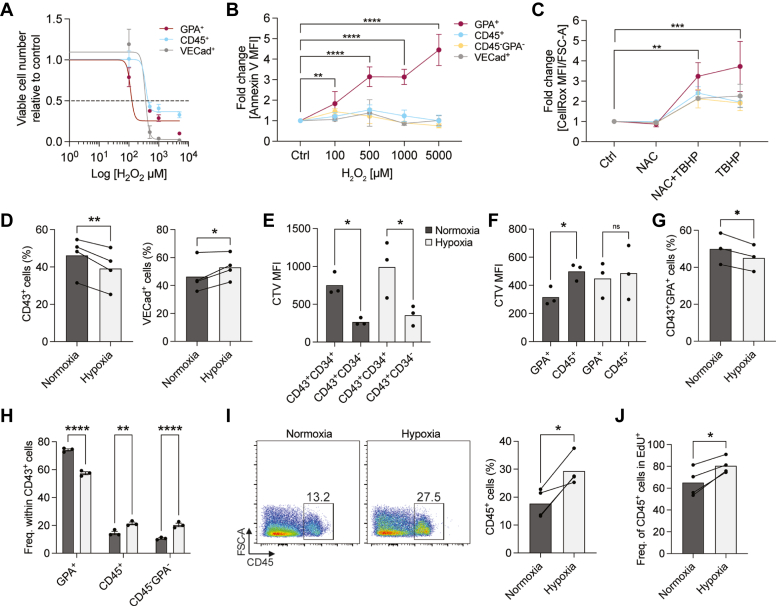
**Oxidative stress impairs erythroid differentiation while hypoxia enhances CD45**^**+**^**cell proliferation.***A*–*C*, iPSC-derived HE cells were subcultured for 6 days (n = 4 biological replicates). *A*, nonlinear regression inhibition curve (inhibition dose-response) representing percentages of viable cells ± SEM after an acute H_2_O_2_ treatment (0, 100, 500, 1000, and 5000 μM) and recovery of HE-derived populations. Parameters were different for at least one data set with *p* value < 0.0001; IC50_GPA+_ = 114.3 μM, IC50_CD45+_ = 363.4 μM, and IC50_VECad+_ = 354.1 μM. *B*, Annexin V MFI levels ± SEM is shown as fold change relative to the control following acute H_2_O_2_ treatment and recovery of HE-derived populations. *C*, MFI of CellRox/FSC-A levels ± SEM is shown as fold change relative to the control, following NAC (2500 μM) and/or tBHP (200 μM) treatment of HE-derived populations. *D*–*G*, iPSC-derived HE cells were subcultured in hypoxia (4% O_2_) or normoxia (ambient) for 3 days. The frequency of CD43^+^ and VECad^+^ cells (n = 4 biological replicates, paired t tests) (*D*), proliferation rates *via* CellTrace Violet (CTV) in early hematopoietic (*E*) and more mature GPA^+^ and CD45^+^ cells (n = 3 biological replicates, ANOVA tests) (*F*), as well as frequency of CD43^+^GPA^+^ cells (n = 3 biological replicates, paired *t* test) (*G*) were assessed by flow cytometry. *H*-*J*, iPSC-derived HE cells were subcultured in hypoxia (4% O_2_) or normoxia (ambient) for 6 days. The frequency of the indicated populations in CD43^+^ cells (n = 3 biological replicates, ANOVA test) (*H*) and the frequency of total CD45^+^ cells as representative flow cytometry plots and bar graphs are shown (n = 3 biological replicates, paired *t* test) (*I*). *J*, proliferation was measured by EdU incorporation and the frequency of CD45^+^ cells in the EdU^+^ population was assessed by flow cytometry at 24 h postpulse (n = 4 biological replicates, paired *t* test). ns, not significant, ∗*p* < 0.05, ∗∗*p* < 0.01, ∗∗∗*p* < 0.001, ∗∗∗∗*p* < 0.0001. HE, hemogenic endothelial; iPSC, induced pluripotent stem cell; MFI, mean fluorescence intensity; NAC, *N*-acetylcysteine; tBHP, tert-Butyl hydroperoxide.

Based on our findings at the transcriptional level, we sought to compare the effects of oxidative stress on the GPA^+^ erythroid population (deriving from MEP) and the CD45^+^ cell population (deriving from GMP). At day six of HE subculture, we exposed the cells to a range of hydrogen peroxide (H_2_O_2_) concentrations between 100 and 5000 μM, followed by a recovery period, before examining their viability and apoptosis levels. Excess H_2_O_2_ is a well-described source of oxidative stress and high levels of H_2_O_2_ lead to cell death (35). Viable cell numbers sharply decreased for GPA^+^, CD45^+^, and VECad^+^ cells with high H_2_O_2_ concentrations, in a dose-dependent manner (Fig. 2*A*). Intriguingly, we observed distinct patterns of viability for these three populations in response to acute H_2_O_2_ treatment. While there were almost no viable VECad^+^ cells left following treatment with 500 μM H_2_O_2_, in contrast, we observed a steadier decline for GPA^+^ and CD45^+^ cells (Fig. 2*A*). Moreover, calculation of a nonlinear regression inhibition curve revealed a significantly lower half-maximal inhibitory concentration (IC50) for GPA^+^ cells (IC50_GPA+_ = 114.3 μM), compared to the other populations (IC50_CD45+_ = 363.4 μM and IC50_VECad+_ = 354.1 μM). Interestingly, among surviving GPA^+^ cells there were higher levels of apoptotic cells as compared to other cell types, and these levels increased in parallel with the dose of H_2_O_2_ (Fig. 2*B*). These results suggest that following acute oxidative stress and a recovery period, surviving CD45^+^ cells overcome cell death more efficiently than GPA^+^ cells. To understand how the level of ROS changes with oxidative stress, we measured ROS levels following treatment with oxidant *tert*-Butyl hydroperoxide (tBHP) with or without *N*-acetylcysteine (NAC, an antioxidant). We found that tBHP led to higher ROS levels in GPA^+^ cells compared to other cell types (Fig. 2*C*), even in the presence of NAC. Taken together, these results show that GPA^+^ cells are more vulnerable to oxidative stress compared to CD45^+^ cells.

### Hypoxic conditions distinctly affect the proliferation of HE-derived hematopoietic lineages

We then set out to understand the effects of hypoxia on the emergence of HE-derived hematopoietic populations. After 3 days in hypoxic conditions, we observed a slight (15.3%) decrease in emerging CD43^+^ hematopoietic cells concomitant with a similar (12.6%) increase in VECad^+^ endothelial cells (Fig. 2*D*). It is known that cell proliferation may be delayed in hypoxic culture conditions compared to cultures in ambient O_2_ levels (36). Therefore, to understand how hypoxia affects emerging hematopoietic cells, we measured the proliferation rate of CD43^+^CD34^+^ early progenitors and more mature CD43^+^CD34^-^ hematopoietic cells. While we observed that CD34^+^ early progenitors proliferated less than hematopoietic cells which have lost CD34 expression, we did not detect changes between hypoxic and normoxic conditions for these early stage populations (Fig. 2*E*). In contrast, when we looked into specific lineages, we found that GPA^+^ erythroid cells proliferated more than CD45^+^ cells in normoxic conditions (Fig. 2*F*), as we have shown previously (15). Intriguingly, this difference was not apparent in hypoxia, and we observed similar proliferative rates for both lineages (Fig. 2*F*). In line with this result, we observed slightly lower frequencies of GPA^+^ cells in hypoxic *versus* normoxic conditions (Fig. 2*G*), explaining the difference seen in Fig. 2*D*.

As most CD45^+^ cells emerge at a later time point compared to GPA^+^ cells, we evaluated the effects of hypoxic conditions on HE subculture on day six. We found that within the CD43^+^ population, the frequency of GPA^+^ cells was decreased, while the frequencies of CD45^+^ cells and CD45^-^GPA^-^ cells were significantly increased (Fig. 2*H*). Moreover, total CD45^+^ cell frequency was significantly increased in hypoxia compared to normoxia (Fig. 2*I*). We suspected that this difference may be due to proliferation and assessed EdU incorporation with a pulse on day five. At 24 h post-pulse on day six, we detected enhanced proliferative rates of CD45^+^ cells in hypoxic conditions (Fig. 2*J*), in contrast to similar rates at day three (Fig. 2*F*). These results show that HE-derived hematopoietic populations respond differently to hypoxic conditions and suggest that GPA^+^ and CD45^+^ cells may have different response mechanisms to oxidative stress.

### Decreased NRF2 activity in hypoxic conditions affect erythroid cell formation

A previous study has shown that peroxidasin expression, which we found to be increased in hypoxia in MEPs (Fig. 1*G*), is directly regulated by the NRF2 transcription factor (37). NRF2 is a master regulator of antioxidant response involved in the cellular defense against ROS and oxidative stress, and stimulates the expression of enzymes producing NADPH and redox cycling enzymes, as well as genes involved in the synthesis and utilization of reduced glutathione (GSH) (38). We have previously shown that chemical inhibition of NRF2 leads to an impaired erythroid differentiation from HE cells (39). Therefore, we hypothesized that if NRF2 is decreased in hypoxia, this may lead to a suboptimal antioxidant response in erythroid cells.

**Figure 3:**
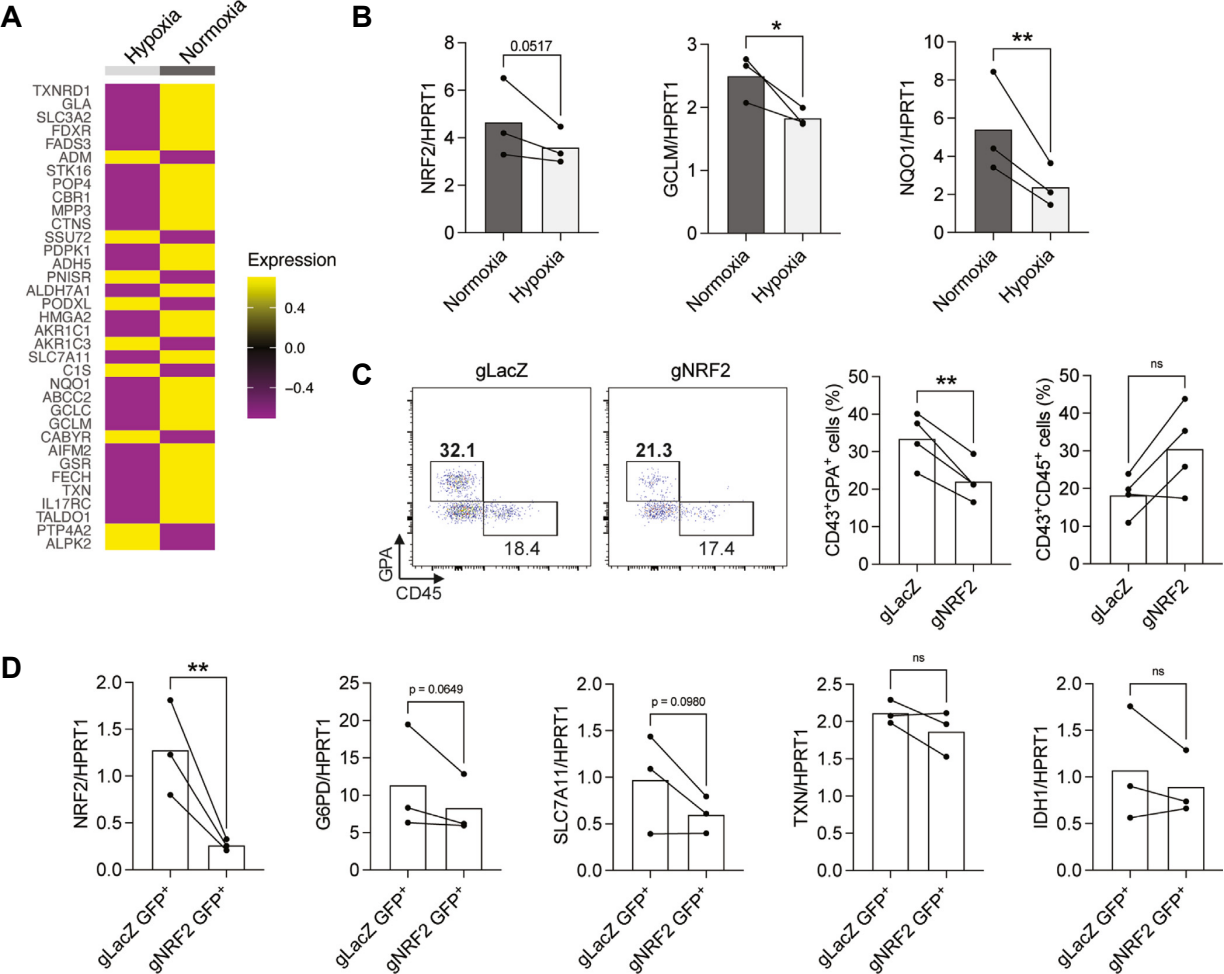
**NRF2 and its targets are altered in hypoxia and erythroid cells are sensitive to NRF2 downregulation.***A*, iPSC-derived HE cells were cultured in hypoxia (4% O_2_) or normoxia (ambient) for 72 h and analyzed by scRNAseq. The average expression of NRF2 biomarker genes in hypoxia *versus* normoxia samples are shown as a heatmap. *B*, bar graphs show transcripts of the indicated genes normalized to *HPRT1* expression (n = 3 biological replicates, paired t tests). *C*–*D*, iPSC-derived HE cells were transduced with GFP-expressing CRISPRi vectors harboring sgRNAs for NRF2 or LacZ (nontargeting control) and cultured for 6 days *C*, the expression of GPA and CD45 within GFP+ cells was assessed by flow cytometry at day six (n = 4 biological replicates, paired t tests). *D*, bar graphs show transcripts of the indicated genes normalized to *HPRT1* expression in sorted GFP+ cells at day six (n = 3 biological replicates, paired t tests). ns, not significant, ∗*p* < 0.05, ∗∗*p* < 0.01. HE, hemogenic endothelial; iPSC, induced pluripotent stem cell; scRNAseq, single-cell RNA sequencing; sgRNA, single guide RNA.

We assessed the expression of NRF2 biomarker genes, compiled by Rooney *et al.* (40) and detected that 35 biomarker genes were expressed in our RNAseq dataset (Fig. 3*A*). Interestingly, 26 genes out of 35 (74%) were decreased in hypoxia compared to normoxia. We confirmed by quantitative polymerase chain reaction that *NRF2*, and its well-described target genes *GCLM* and *NQO1* were decreased in hypoxia *versus* normoxia (Fig. 3*B*). To further validate our previous findings on NRF2 requirement for erythroid differentiation, we knocked down NRF2 expression using CRISPRi and observed an impaired formation of erythroid cells, in contrast to CD45^+^ cells which did not show significant changes (Fig. 3*C*). We showed by quantitative polymerase chain reaction that *NRF2* was significantly downregulated and the expression of two of its targets, *G6PD* and *SLC7A11*, were slightly decreased following CRISPRi, while other targets *TXN* or *IDH1* were not affected (Fig. 3*D*). These results show that hypoxia diminishes NRF2-mediated antioxidant response, and while HE-derived erythroid cell formation is altered by NRF2 downregulation, CD45^+^ cells are not affected.

### Low GSH levels and high lipid peroxidation predispose erythroid cells to oxidative stress

**Figure 4:**
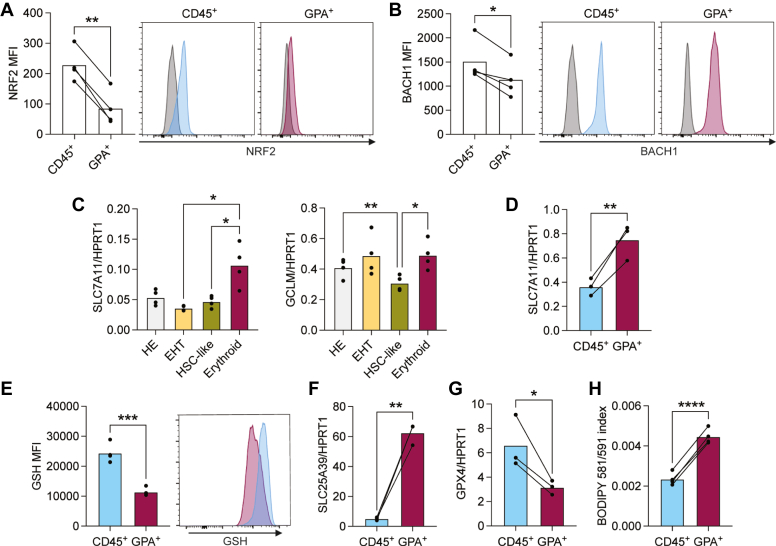
**The glutathione pathway is upregulated and GSH is imported into mitochondria in erythroid cells.***A*-*B*, iPSC-derived HE cells were subcultured for 6 days. NRF2 (*A*) and BACH1 (*B*) protein levels were assessed by intracellular staining and detected by flow cytometry. Bar graphs and representative flow cytometry plots are shown (n = 4 biological replicates, paired t tests). *C*, bar graphs show transcripts of the indicated genes normalized to *HPRT1* expression in the indicated sorted populations from day eight EBs (n = 4 biological replicates, ANOVA test). *D*–*G*, iPSC-derived HE cells were subcultured for 6 days. *D*, bar graphs show *SLC7A11* transcripts normalized to *HPRT1* expression in sorted CD45^+^ or GPA^+^ cells (n = 3 biological replicates, paired *t* test). *E*, GSH levels were detected with ThiolTracker Violet by flow cytometry, in CD45^+^ or GPA^+^ cells (n = 3 biological replicates, paired t tests). *F*, bar graph shows *SLC25A39* transcripts normalized to *HPRT1* expression in sorted CD45^+^ or GPA^+^ cells (n = 3 biological replicates, paired *t* test). *G*, bar graph shows *GPX4* transcripts normalized to HPRT1 expression in sorted CD45^+^ or GPA^+^ cells (n = 3 biological replicates, paired *t* test). *H*, lipid peroxidation was detected using the BODIPY 581/591 C11 Sensor, which shifts excitation and emission maxima from 581/591 nm to 488/510 nm after oxidation. Bar graphs show ratiometric measurement of lipid peroxidation (n = 4 biological replicates, paired *t* test). ∗*p* < 0.05, ∗∗*p* < 0.01, ∗∗∗*p* < 0.001, ∗∗∗∗*p* < 0.0001. EB, embryoid body; GSH, glutathione; HE, hemogenic endothelial; iPSC, induced pluripotent stem cell.

As we observed that hypoxia or NRF2 downregulation induces a deleterious effect on expression of genes involved in GSH synthesis (*SLC7A11*, *GSR*, *GCLC*, *GCLM*, and *SLC3A2*; Fig. 3, *A*, *B* and *D*), we further investigated the role of this pathway in HE-derived hematopoiesis. GSH is an essential antioxidant formed by the combination of glutamate, glycine, and cysteine (41). It is a crucial player in scavenging ROS and can directly act against free radicals and prooxidants, or as a cofactor for the glutathione peroxidase family (GPx). The GSH-GPX4 pathway is activated or suppressed by NRF2 and BACH1, respectively (42). The distinct effects of NRF2 downregulation in GPA^+^ *versus* CD45^+^ cells prompted us to investigate protein levels of NRF2 and BACH1 in these cells. We found that CD45^+^ cells express higher levels of NRF2 compared to GPA^+^ cells (Fig. 4*A*), in line with the increased sensitivity to oxidative stress and the drastic effect of NRF2 downregulation in GPA^+^ cells. Of note, we did not detect a significant difference in GPA^+^ *versus* CD45^+^ cells in the levels of NFE2 (Fig. S2*A*), which was shown to compete with NRF2 to bind its target genes (43).

On the other hand, we found that BACH1 levels were slightly higher in CD45^+^ cells compared to GPA^+^ cells (Fig. 4*B*), which may lead to a decrease in glutathione pathway activity in these cells.

Notably, *SLC7A11*, responsible for the transport of cystine, and *GCLC*, which catalyzes the rate limiting step in GSH production, had higher expression levels in erythroid (GPA^+^) cells compared to immunophenotypic HSC-like cells at day eight of iPSC-derived hematopoietic differentiation (Fig. 4*C*).

Since we have seen that GPA^+^ and CD45^+^ presented different responses to hypoxia and NRF2 downregulation, we compared the role of GSH metabolism specifically in these two populations. Expression of *SLC7A11* and *GCLC*/*GCLM* (glutamate–cysteine ligase) were higher in GPA^+^ cells compared to CD45^+^ cells (Fig. 4*D* and S2*B*), in line with the lower BACH1 levels we detected in CD45^+^ cells. However, surprisingly, intracellular GSH levels were lower in GPA^+^ cells than in CD45^+^ cells (Fig. 4*E*). Recently, erythroid cell development was found to rely on the import of GSH into mitochondria by the SLC25A39 transporter in mice (44). We therefore assessed the expression level of *SLC25A39* and found that GPA^+^ cells showed significantly higher expression compared to CD45^+^ cells (Fig. 4*F*). These results suggest that GSH may be transported into mitochondria in erythroid cells, limiting its use for combatting oxidative stress in the cytosol.

The high GSH levels and better response to oxidative stress we have observed in CD45^+^ cells in comparison to GPA^+^ cells prompted us to investigate lipid peroxidation levels in these cells. Previously, it was found that HSCs decrease their lipid peroxidation levels *via* the hydroperoxide scavenger GPX4, which uses GSH as a cofactor (45). Moreover, it is known that erythrocytes are susceptible to lipid peroxidation due to their specific lipid composition and their role as oxygen carriers (46). Indeed, we detected a higher expression of the scavenger *GPX4* in CD45^+^ cells (Fig. 4*G*), along with lower levels of lipid peroxidation as compared to GPA^+^ cells (Fig. 4*H*), explaining how CD45^+^ cells withstand oxidative stress better than GPA^+^ cells.

### HE-derived CD45^+^ cells preferentially use the noncanonical TCA cycle

**Figure 5:**
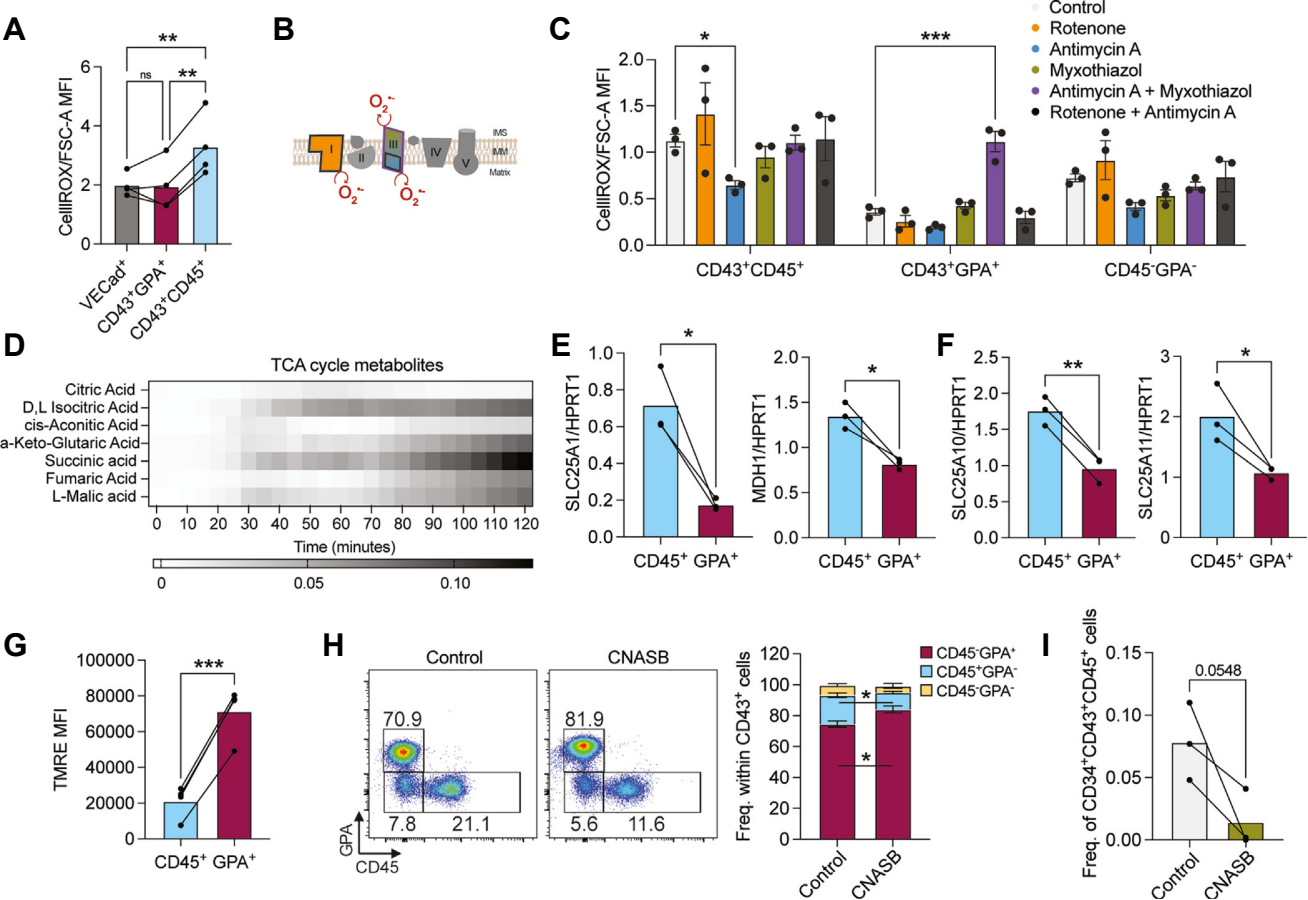
**The use of the noncanonical TCA cycle is essential for CD45**^**+**^**cells.***A*, MFI of CellRox/FSC-A levels is shown in HE-derived populations, following 6 days of subculture (n = 4 biological replicates, ANOVA test). *B*, scheme of the electron transport chain (ETC) with complexes I to V. Sites where ROS is produced are shown in *red*. Color code denotes the targets of the compounds indicated in (*C*). IMS = intermembrane space, IMM = inner mitochondrial membrane. *C*, iPSC-derived HE cells were subcultured for 6 days, collected, and incubated with myxothiazol (1.5 μM), antimycin A (40 μM) and/or rotenone (1 μM). MFI of CellRox/FSC-A levels is shown in HE-derived populations (n = 4 biological replicates, ANOVA test). *D*, mitochondrial electron flow assay (MitoPlate S-1) was performed on subcultured HE cells at 48 h, to detect TCA cycle substrates that are used to fuel mitochondrial activity. Heatmap shows average values of n = 3 biological replicates). *E*-*I*, iPSC-derived HE cells were subcultured for 6 days. *E*–*F*, bar graphs show transcripts of the indicated genes normalized to *HPRT1* expression in sorted CD45^+^ or GPA^+^ cells (n = 3 biological replicates, paired t tests). *G*, mitochondrial membrane potential was assessed *via* TMRE by flow cytometry in CD45^+^ or GPA^+^ cells (n = 4 biological replicates, paired t tests). *H*–*I*, HE cells were subcultured for 6 days with or without 200 μM CNASB (SLC25A1 inhibitor). The frequency of the indicated hematopoietic populations in CD43^+^ cells as representative flow cytometry plots and bar graphs (n = 3 biological replicates, ANOVA tests) (*H*), as wells as the frequency of early CD45^+^ progenitors (n = 3 biological replicates, paired *t* test) (*I*), assessed by flow cytometry, are shown. ns, not significant, ∗*p* < 0.05, ∗∗*p* < 0.01, ∗∗∗*p* < 0.001. HE, hemogenic endothelial; iPSC, induced pluripotent stem cell; MFI, mean fluorescence intensity; ROS, reactive oxygen species; TCA, tricarboxylic acid; TMRE, tetramethylrhodamine, ethyl ester.

Interestingly, endothelial (VECad^+^) and GPA^+^ cells had similar ROS levels, while CD45^+^ cells had higher levels compared to both populations (Fig. 5*A*). As we found that erythroid cells are more sensitive to oxidative stress than CD45^+^ cells, we hypothesize ROS levels may be more regulated in GPA^+^ cells and maintained at low levels. In the electron transport chain (ETC), ROS generated from complex I and complex III (Q_i_ or Q_o_ sites) (Fig. 5*B*) may lead to oxidative stress (47). To understand where ROS is produced in HE-derived hematopoietic populations, we treated cells with rotenone (complex I inhibitor), antimycin A (complex III Q_i_ inhibitor), or myxothiazol (complex III Q_o_ inhibitor).

In both CD45^+^ and GPA^+^ cells, antimycin A treatment leads to a decrease in ROS levels compared to the controls, suggesting that complex III Q_i_ site produces ROS in both cell types (Fig. 5*C*). In contrast, myxothiazol treatment did not change ROS levels and therefore complex III Q_o_ site does not seem to contribute to ROS production in these cells. Intriguingly, blocking both Q_i_ and Q_o_ sites of complex III with antimycin A/myxothiazol leads to a three-fold increase in ROS levels, in erythroid cells only (Fig. 5*C*). This high ROS production from complex I can result from reverse electron transport (RET), whereby electrons move from complex II to complex I in cases where high levels of succinate are present (48). We therefore measured the use of TCA cycle metabolites as fuels for the ETC in HE-derived cells after 5 days of subculture (>70% erythroid cells) and detected the highest signal with succinate as a substrate (Fig. 5*D*). Taken together, these results suggest that due to high succinate levels, erythroid cells are prone to generating ROS *via* RET, which could be detrimental to these cells as their intracellular GSH levels are low due to mitochondrial import (Fig. 4*E*, SF).

The distinct ETC activity we observed between erythroid and nonerythroid populations prompted us to further investigate the use of the TCA cycle in these cells. We previously showed that in HE cells, a boost in the TCA cycle with dichloroacetate preferentially diverts these cells to the CD45^+^ lineage, due the use of acetyl-coA for cholesterol synthesis, following citrate export from mitochondria (16). As citrate export is mediated by SLC25A1, we further investigated this pathway. Recently, a noncanonical TCA cycle dependent on SLC25A1 and cytosolic conversion of oxaloacetate to malate by MDH1 was described in highly proliferative embryonic stem cells (49). We found that both *SLC25A1* and *MDH1* were expressed at significantly higher levels in CD45^+^ cells compared to GPA^+^ cells (Fig. 5*E*). Moreover, malate importers *SLC25A10* and *SLC25A11* were also expressed at higher levels in CD45^+^ cells (Fig. 5*F*), suggesting that CD45^+^ cells may be preferentially using the noncanonical TCA cycle, unlike GPA^+^ cells. The use of the alternative TCA cycle leads to less NADH production in mitochondria which can limit OXPHOS (49). Indeed, we observed lower mitochondrial activity in CD45^+^ cells as compared to GPA^+^ cells (Fig. 5*G*).

To test whether CD45^+^ cells depend on the noncanonical TCA cycle, we treated HE cells with a SLC25A1 inhibitor, CNASB, for 6 days. Blocking SLC25A1 with CNASB significantly decreased the frequency of CD45^+^ cells in the CD43^+^ population and skewed the hematopoietic output toward erythroid cells (Fig. 5*H*). Moreover, we found that CNASB treatment led to an 80% decrease in the formation of early CD45^+^ progenitors that still express CD34^+^ hematopoietic progenitor marker (Fig. 5*I*). Taken together, these results show that while GPA^+^ cells use the canonical TCA cycle which is capable of replenishing their succinate and NADH levels, in contrast, CD45^+^ cells rely on the alternative TCA cycle to fuel their cell type-specific metabolic requirements.

## Discussion

In this study, using human iPSC-derived HE cells, we investigated the metabolic differences between HE-derived hematopoietic populations following their commitment to specific lineages. As HE and newly emerging hematopoietic cells reside in hypoxic conditions in the embryo, we assessed the effect of hypoxic conditions on iPSC-derived HE cells and found that MEP cells expressed genes related to oxidative stress at increased levels in these conditions. Previous studies in mice have shown that knocking down *Hif1a* leads to defects in erythroid development, with significantly reduced burst-forming unit—erythroid in the YS at E10 (18), and MPPs isolated from *Hif1a*^−/−^ YS present impaired erythroid colony formation and proliferative defect, affecting erythroid expansion and terminal differentiation (50). Similarly, in humans, when cord-blood hematopoietic stem and progenitor cells are cultured in 1% O_2_, erythroid progenitors are stuck at the burst-forming unit—erythroid stage and the expression of the erythroid marker CD36 is inhibited (51). In addition to these findings, we and others have shown that late-stage erythropoiesis is especially sensitive to oxidative stress (52, 53) and at the same time oxidative stress response is critical and highly regulated in red blood cells (54). Taken together, these results suggest that in hypoxic conditions, HE-derived erythroid cells activate mechanisms to counteract oxidative stress.

We show here that hypoxia leads to an impairment in NRF2-regulated genes and that NRF2 is essential for HE-derived erythroid cell formation, though the regulation of antioxidant responses including GSH metabolism. It has been previously shown that NRF2 protects erythroid cells against oxidative stress by inducing EZH2 and ATF4 *via* miR-214 repression (55). Additionally, ATF4 and integrated stress response were previously linked to the induction of hypoxic stress-responsive factors (56). We show here that the expression of NRF2 and its target genes are affected by hypoxia and propose that the sensitivity of HE-derived erythroid cells to oxidative stress is influenced by this alteration.

We provide evidence that erythroid cells express high levels of genes involved in GSH metabolism, such as SLC7A11 and GCLC/GCLM. Previously, cells with high levels of SLC7A11 were shown to present an increased sensitivity to H_2_O_2_ due to NADPH depletion (57). Indeed, we observed that GPA^+^ cells (with high SLC7A11 levels) were especially sensitive to acute H_2_O_2_ treatment, showing a sharp decline in viability and increase in apoptotic cells as compared to CD45^+^ cells which express lower levels of SLC7A11. Moreover, we find that cytoplasmic GSH levels are low in erythroid cells, most likely due to mitochondrial import of GSH *via* SLC25A39. The SLC25A39 transporter was described to play an important role in erythropoiesis in previous studies (44, 58), by shuttling GSH into mitochondria. The SLC25A39-mediated shuttling of GSH has been shown to support Fe-S cluster biogenesis, iron homeostasis, as well as mitochondrial OXPHOS (44, 59, 60). These results are in line with the elevated mitochondrial activity we have observed in HE-derived GPA^+^ cells.

In stark contrast to erythroid cells, HE-derived CD45^+^ cells express higher levels of *HIF1A*, showed increased proliferation in hypoxia and were not affected by NRF2 downregulation, suggesting that these cells exhibit different response mechanisms to oxidative stress. In line with our findings, HIF1A was found to be upregulated in CD45^+^ cells derived from human embryonic stem cells in hypoxic conditions, and hypoxia was found to play an important role in CD45^+^ T lineage hematopoiesis (61, 62). We also show here that HE-derived CD45^+^ cells have higher ROS levels compared to GPA^+^ cells. Interestingly, a previous study has shown that ROS (H_2_O_2_) blocks pyruvate dehydrogenase kinase and enhances pyruvate use for the TCA cycle (63). As we have previously shown that boosting pyruvate flow into the TCA cycle by dichloroacetate increases CD45^+^ cell formation through the mevalonate pathway (16), ROS may play a role in CD45^+^ cell specification. Moreover, the mevalonate pathway was shown to control GPX4 synthesis and thereby prevent ferroptosis (64). High levels of GPX4 were found to protect against lipid peroxidation (65), and we show here that HE-derived CD45^+^ cells express higher GPX4 levels along with lower levels of lipid peroxidation, as compared to GPA^+^ cells. In line with this, it has been shown that knockdown of GPX4 does not affect early stages of human hematopoietic stem and progenitor cell-derived erythroid differentiation (66). As we found that lipid peroxidation levels are higher in erythroid cells, it is probable that these cells undergo cell death through ferroptosis (67); however, further studies are needed to elucidate this mechanism.

Hypoxic conditions induce sharp increases in ROS production, leading to damages in mitochondria (68). Interestingly, the increased ROS in hypoxia results from the mitochondrial complex III in particular (17), which we have found to produce ROS in both GPA^+^ and CD45^+^ HE-derived hematopoietic cells. Moreover, we describe here that increased levels of succinate in erythroid cells may predispose them to the activation of RET that induces ROS production from mitochondrial complex I. A previous study has shown that hypoxia leads to an increase in the use of succinate and activates RET-mediated ROS production from complex I (69). This study also demonstrates that a concomitant increase in NADH levels is required to induce a response to hypoxia. Together, these findings suggest that the sensitivity of erythroid cells to hypoxia-related oxidative stress may partially result from an increased production of ROS from complex I fueled by succinate. Indeed, we show here that succinate is the main fuel for the ETC in erythroid cells.

In contrast, we find that HE-derived CD45^+^ cells rely on the noncanonical TCA cycle, by which citrate is exported *via* SLC25A1 to the cytoplasm, converted to oxaloacetate and then malate, and taken up by mitochondria to complete the partial cycle. In this alternative TCA cycle, succinate production is decreased (49) and this is in line with our results showing that CD45^+^ cells do not exhibit RET when complex III is blocked.

Taken together, our results show that HE-derived GPA^+^ cells use GSH for mitochondrial processes and rely on NRF2 to counteract hypoxia and oxidative stress. On the other hand, HE-derived CD45^+^ cells are more resistant to ROS (including lipid peroxidation) and show increased proliferation in hypoxia, due to their preferential use of noncanonical TCA cycle diminishing ROS production from complex I. Our findings underline the contribution of hypoxia and oxidative stress to defining cell identities and elucidate the strictly different metabolic needs of HE-derived hematopoietic populations.

## Experimental procedures

### Human iPSC culture and hemogenic endothelium differentiation

The human iPS cell line RB9 (70) was cocultured with mitotically inactivated mouse embryonic fibroblasts in Dulbecco's modified Eagle's medium/F-12 supplemented with 20% KnockOut-serum replacement, glutamax, nonessential amino acids, 50 μM beta-mercaptoethanol, and 10 ng/ml fibroblast growth factor in a humidified incubator at 37 °C, 5% CO_2_. Cells were routinely tested for *mycoplasma* and confirmed to be free from contamination. Hematopoietic differentiation was induced as described by Ditadi *et al.* (71) with modifications indicated in Oburoglu *et al.* (16). Briefly, six days after passaging, embryoid bodies (EBs) were formed by lifting iPSC colonies with dispase and cultured in ultra-low attachment flasks. Between days 0 to 3, the EBs were kept in SFD medium (71) with 1 ng/ml activin A. On day two, the medium was supplemented with 3 μM CHIR99021. Between days three to eight, the medium was changed to SP34 medium (71). On day three, the medium was supplemented with 1 ng/ml activin A and 3 μM CHIR99021. Between days six to eight, the medium was supplemented with 200 ng/ml SCF, 4 IU EPO, 50 ng/ml insulin-like growth factor I, 20 ng/ml interleukin 6 (IL-6), and 10 ng/ml IL-11. On day eight, EBs were dissociated to obtain a single-cell suspension. CD34^+^ cells were selected using the human CD34 MicroBead kit (Miltenyi Biotec) and immediately frozen in CryoStor cryopreservation medium.

### HE cell culture

CD34^+^ HE cells were thawed in RPMI medium with 1% penicillin–streptomycin and 10% fetal calf serum. This medium was washed away and cells were plated (60,000–100,000 cells per well) on rhLaminin-521-coated flat bottom 96-well plates in HE medium (71) with ROCK-inhibitor (Y-27632) and kept in a humidified incubator at 37 °C, 5% CO_2_, and 4% O_2_ overnight. The following day (day 0), the cells were carefully washed twice with PBS, and fresh HE medium was added. Where indicated, media was supplemented with 200 μM CNASB (4-Chloro-3-[[(3-nitrophenyl)amino]sulfonyl]-benzoic acid) (72). Cells were kept in a humidified incubator at 37 °C in normoxia (5% CO_2_ and 20% O_2_) or hypoxia (5% CO_2_ and 4% O_2_) with media changes every 3 days.

### Flow cytometry analyses

Cells were collected after a 2-min incubation at 37 °C with StemPro Accutase Cell Dissociation Reagent, washed with PBS 2% fetal calf serum and stained with flow cytometry antibodies. Fluorescence was measured on a BD FACSymphony A1 or a BD LSRFortessa. Flow cytometry outputs were analyzed on FlowJo Software (www.flowjo.com), with initial gatings on SSC-A/FSC-A, FSC-H/FSC-A, SSC-H/SSC-A, and 7-AAD for doublet and dead cell exclusion. Gating strategies for the different lineage were as follows: GPA^+^ cells (VECad^-^CD43^+^CD45^-^GPA^+^), CD45^+^ cells (VECad^-^CD43^+^CD45^+^GPA^-^), CD45^-^GPA^-^ cells (VECad^-^CD43^+^CD45^-^GPA^-^), and VECad^+^ cells (VECad^+^CD43^-^).

To measure proliferation, cells were assayed with CellTrace Violet with a 10-min incubation according to the manufacturer’s instructions. To measure proliferation by EdU incorporation, cells were given a 24-h EdU pulse before using Click-iT EdU Flow Cytometry Cell Proliferation Assay, according to the manufacturer’s instructions. To measure NRF2, NFE2, and BACH1 transcription factor levels, following surface staining with antibodies against GPA, CD45, and Live/Dead fixable stain, cells were fixed and permeabilized using the True-Nuclear Transcription Factor Buffer Set, according to the manufacturer’s protocol. Cells were then stained with anti-NRF2-AF488, anti-NFE2, or anti-BACH1 antibodies. The anti-NFE2 and anti-BACH1 antibodies were detected using a donkey anti-rabbit AF488 secondary antibody. To measure lipid peroxidation, cells were incubated with 0.5 μM BODIPY 581/591 C11 Lipid Peroxidation Sensor, according to the manufacturer’s instructions. The ratio of mean fluorescence intensity 581/mean fluorescence intensity 591 was calculated by flow cytometry, as described previously (73). To measure intracellular ROS levels, cells were incubated with 2500 μM NAC (negative control), and/or 200 μM tBHP (positive control) for 1 h; or with 1.5 μM myxothiazol, 40 μM antimycin A, and/or 1 μM rotenone for 1 h before incubation with CellROX Green Reagent according to the manufacturer’s instructions. To measure response to H_2_O_2_ and cell vulnerability to oxidative stress, cells were incubated with 0, 100, 500, 1000, or 5000 μM H_2_O_2_ for 1 h, followed by a 2-h recovery in HE medium. Cells were collected and apoptosis was detected with the Annexin V Apoptosis Detection Kit (PE) according to manufacturer’s instructions. Viable cells were assessed with a nonlinear regression inhibition curve (inhibition dose-response) with fixed hill slope, using three parameters (IC50, Top, Bottom) and concluded that three parameters were different for at least one data set with *p* value < 0.0001. To measure GSH levels, cells were incubated with ThiolTracker Violet according to the manufacturer’s instructions. For all aforementioned assays, fluorescence was measured simultaneously with hematopoietic lineage markers, as indicated.

### NRF2 knockdown *via* CRISPRi

Single guide RNA (sgRNA) sequences (Table 3) for LacZ and NRF2 were cloned into the GFP-expressing pLV hU6-sgRNA hUbC-dCas9-KRAB-T2a-GFP vector (74) behind the U6 promoter, as described previously (75). Lentiviral particles were produced in HEK 293T cells. The efficiency of each sgRNA was measured by lentiviral transduction of K562 cells and assessment of corresponding gene expression in GFP+ cells on day three. For NRF2 knockdown in HE cells, the cells were transduced with lentiviral particles on the day after thawing (day 0).

**Table 3:** CRISPRi sgRNA sequences

Gene name	Forward sequence	Reverse sequence
LacZ	CACCGTGCGAATACGCCCACGCGAT	AAACATCGCGTGGGCGTATTCGCAC
NFE2L2 (NRF2)	CACCGGGACAGGGCGGCTCTGGTGG	AAACCCACCAGAGCCGCCCTGTCCC

### Gene expression by real-time quantitative polymerase chain reaction

For comparisons between hematopoietic lineages, the GPA^+^ or CD45^+^ cells were sorted on a BD FACSAria III on day six of HE subculture. For comparisons between hypoxia and normoxia conditions, total HE-derived cells were assessed on day six of HE subculture. For comparisons between EHT subsets, HE (CD34^+^CD43^-^CXCR4^-^CD73^-^CD90^+^VECad^+^), EHT (CD34^+^CD43^int^CXCR4^-^CD73^-^CD90^+^VECad^+^), HSC-like (CD34^+^CD43^+^CD90^+^CD38^−^), and erythroid cells (CD43^+^GPA^+^) were sorted on a BD FACSAria III on day eight of hematopoietic differentiation. Purity checks were performed for all sorted cells. Isolated cells were frozen in RLT buffer (Qiagen) with β-mercaptoethanol, and total RNA was extracted using RNeasy Micro Kit (Qiagen) according to the manufacturer’s instructions. RNA concentrations were determined using a UV spectrophotometer (NanoDrop) and reverse transcription was performed using random hexamer priming and Superscript III (Invitrogen) in Bio-Rad T100 Thermal Cycler. TaqMan assays were used for quantitative real-time PCR analysis on a QuantStudio 1 Real-Time PCR System (Thermo Fisher Scientific), and HPRT1 was used as the reference gene to normalize data.

### Mitochondrial electron flow assay

CD34^+^ HE cells were thawed, plated onto Matrigel (16 μg/cm^2^)-coated 96-well flat bottom plates (60,000–100,000 cells per well) in HE medium with ROCK-inhibitor (Y-27632), and kept in a humidified incubator at 37 °C, 5% CO_2_, and 4% O_2_ overnight. Media were changed every day and the MitoPlate S-1 assay (Biolog) was performed on day five of subculture, according to the manufacturer’s instructions. Substrates of the MitoPlate-S1 wells were transferred onto the cell culture plate and absorbance was read at *A*_590_ at 37 °C every 5 min for 2 h to detect substrate use to fuel mitochondrial activity.

### Nuclei isolation, single-cell library preparation, and sequencing

CD34^+^ HE cells were subcultured on rhLaminin-521-coated flat bottom 48-well plates (100,000–130,000 cells per well) as indicated above, in a humidified incubator at 37 °C in normoxia (5% CO_2_ and 20% O_2_) or hypoxia (5% CO_2_ and 4% O_2_). On day three of subculture, nuclei were isolated following the low nuclei input protocol by 10× Genomics (with 10-min centrifugations). The diluted nuclei buffer, the wash buffer and the lysis buffer were freshly prepared according to manufacturer’s instructions. The lysis buffer used at 0.5× for 2 min for all conditions. In all conditions, we confirmed 100% lysis by microscopy. The Chromium Next GEM Single Cell Multiome ATAC + Gene Expression Reagent Bundle (10× Genomics) was used to prepare the samples according to the manufacturer’s instructions. A Chromium iX was used to do the droplet encapsulation. An Agilent TapeStation and a Qubit Flex were used to generate complementary DNA and library traces and to measure concentrations. Libraries were sequenced on a NovaSeq 6000 according to the recommendations by 10× Genomics with the aim to reach 50.000 reads/nucleus.

### Bioinformatics analysis

The single-cell RNAseq data were processed and analyzed using the Seurat package (Supplementary Resources Table) (76). To identify cells to exclude, we used the following quality control metrics: library size, number of expressed features, and proportions of mitochondrial and ribosomal genes per cell. Cell filtering was performed by thresholding according to parameter values of nreads, ngenes, and perc_mito. Gene filtering was performed by excluding genes that are lowly or not expressed in our system (conserved fraction: 0.89–0.92). To eliminate batch effects, clusters were assigned to cell types individually by cluster annotation in each sample. Cell types were determined based on previously published findings (20, 21, 22, 23, 24). Each sample was analyzed separately using the sctransform method from the Seurat package. We identified mutual nearest neighbors across samples, filtered anchors, and integrated samples using the ssSTACAS method (77). The resulting integrated dataset was used to calculate a UMAP, and downstream analyses were conducted using the Seurat package on R (Supplementary Resources Table). Gene set enrichment analysis (msigdb_v2023.2.Hs) was performed on R with the fgsea package (Supplementary Resources Table) and using hallmark pathways (h.all.v2023.2.Hs).

The scATAC-seq data were submitted to the following quality control analyses: nucleosome banding pattern, transcriptional start site (TSS) enrichment score (ENCODE project: https://www.encodeproject.org/data-standards/terms/), total number of fragments in peaks, fraction of fragments in peaks (cells with low values (*i.e.* <15–20%) were removed) and ratio reads in genomic blacklist regions (ENCODE). We inspected the TSS enrichment scores by grouping the cells based on the score and plotting the accessibility signal over all TSS sites. We used Signac (78) to perform term frequency-inverse document frequency normalization, followed with feature selection (top 50% most common peaks) and singular value decomposition. The HIF1A regulon was determined based on the DoRothEA gene regulatory network as described previously (32).

### Statistical analysis

Experiments were analyzed using GraphPad Prism 10 (Supplementary Resources Table). Tests for statistical significance are indicated in the figure legends. The DEGs were determined between conditions using the Wilcoxon Rank Sum test and genes with at least 0.25-fold difference (log-scale) and an adjusted *p*-value of less than 0.05 were declared as significant.

## Data availability

The 10× Multiome (scRNAseq + scATAC-seq) data presented in this paper are available in the GEO repository under the accession number GSE270141.

## Supporting information

This article contains supporting information.

## Conflict of interest

The authors declare that they have no conflicts of interest with the contents of this article.

## Supporting information

Supplemental Information

Supplemental ResourcesTable
