# Supplementary material for: Hematopoietic cells emerging from hemogenic endothelium exhibit lineage-specific oxidative stress responses
Source: J Biol Chem. 2024 Sep 24;300(11):107815. doi: 10.1016/j.jbc.2024.107815 (PMC11532904; doi:10.1016/j.jbc.2024.107815)
Supplement: Supplemental Information [file mmc1.pdf]

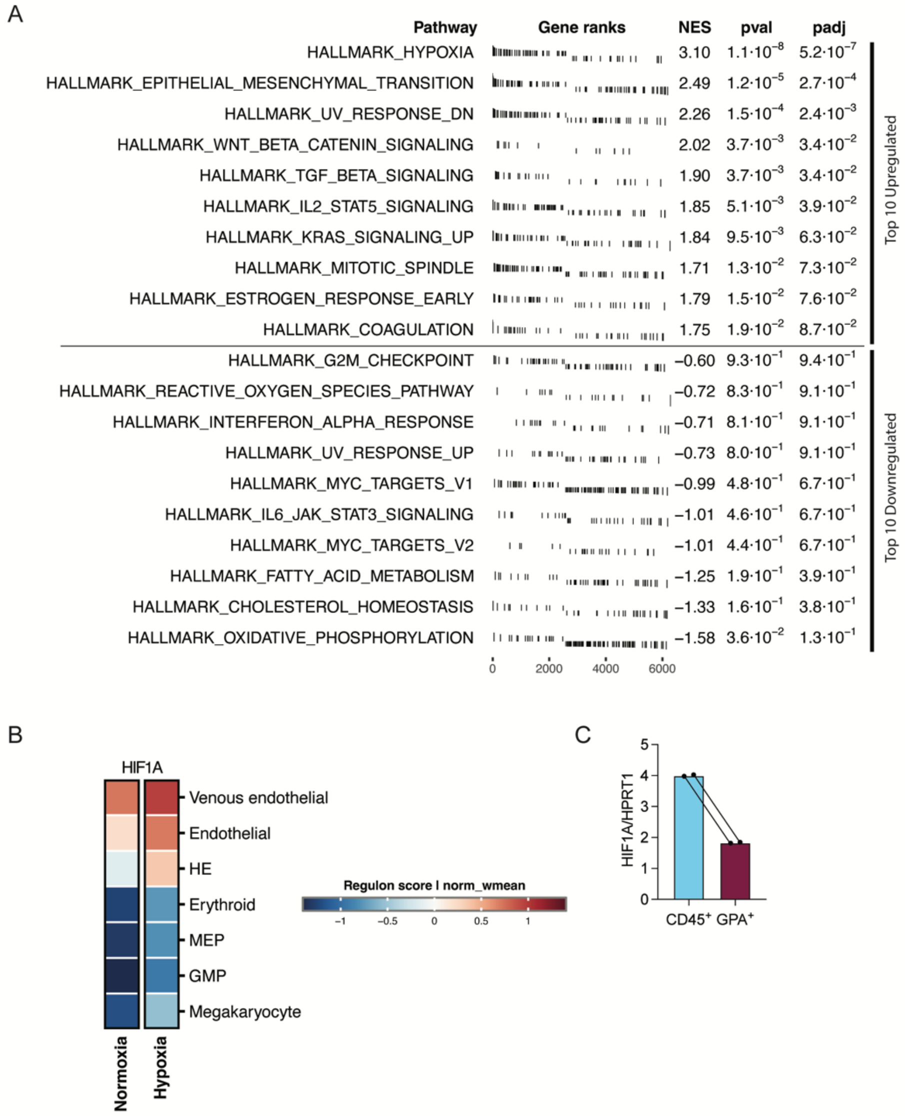

Figure S1. Hypoxic conditions induce transcriptional changes in hematopoietic populations

(A-B) iPSC-derived HE cells were cultured in hypoxia (4% O_2) or normoxia (ambient) for 72h and analyzed by scRNAseq. **(A)** Differentially expressed genes (DEGs) in hypoxia as compared to normoxia were determined by gene set enrichment analysis. The top 10 upregulated or downregulated pathways are shown. **(B)** HIF1A regulon generated by transcription factor-transcriptional targets analysis with DoRothEA is shown for all clusters. **(C)** Bar graphs show *HIF1A* transcripts normalized to *HPRT1* expression in sorted CD45$^+$ or GPA$^+$ cells, following a 6-day subculture of HE cells (n = 2 biological replicates).

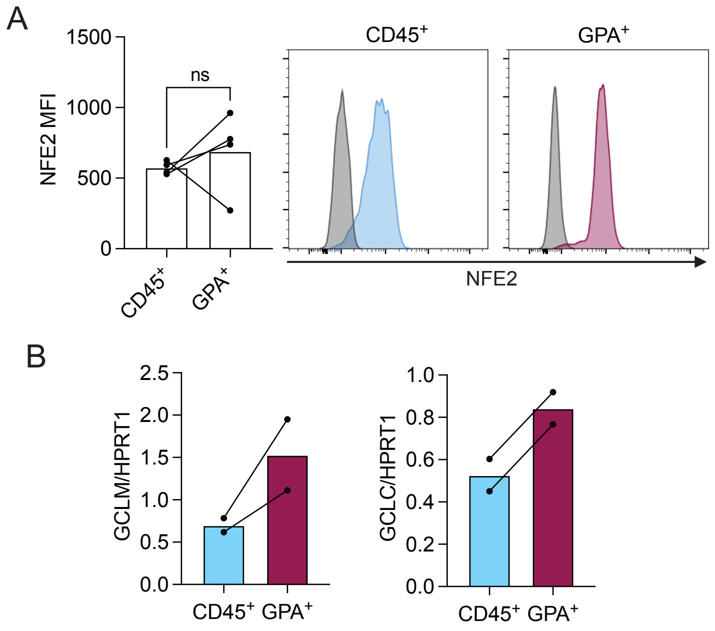

Figure S2. Expression levels of NFE2 and glutamate–cysteine ligase subunits in hematopoietic populations

(A) iPSC-derived HE cells were subcultured for 6 days. NFE2 protein levels were assessed by intracellular staining and detected by flow cytometry. Bar graphs and representative flow cytometry plots are shown (n = 4 biological replicates, paired t test). **(B)** Bar graphs show *GCLM* and *GCLC* transcripts normalized to *HPRT1* expression in sorted $CD45^+$ or GPA^+ cells, following a 6-day subculture of HE cells (n = 2 biological replicates). ns = not significant.
