# Supplementary material for: Hematopoietic cells emerging from hemogenic endothelium exhibit lineage-specific oxidative stress responses
Source: J Biol Chem. 2024 Sep 24;300(11):107815. doi: 10.1016/j.jbc.2024.107815 (PMC11532904; doi:10.1016/j.jbc.2024.107815)
Supplement: Supplemental ResourcesTable [file mmc2.docx]

**RESOURCES TABLE**

| **REAGENT or RESOURCE** | **SOURCE** | **IDENTIFIER** |
| --- | --- | --- |
| **Taqman probes** |  |  |
| NRF2 (NFE2L2) | Thermo Fisher Scientific | Hs00975961_g1 |
| HIF1A | Thermo Fisher Scientific | Hs00153153_m1 |
| GCLC | Thermo Fisher Scientific | Hs00155249_m1 |
| GCLM | Thermo Fisher Scientific | Hs00978072_m1 |
| NQO1 | Thermo Fisher Scientific | Hs01045993_g1 |
| G6PD | Thermo Fisher Scientific | Hs00166169_m1 |
| TXN | Thermo Fisher Scientific | Hs01555214_g1 |
| IDH1 | Thermo Fisher Scientific | Hs04966975_g1 |
| SLC7A11 | Thermo Fisher Scientific | Hs00921938_m1 |
| GPX4 | Thermo Fisher Scientific | Hs00989766_g1 |
| SLC25A39 | Thermo Fisher Scientific | Hs00924971_g1 |
| SLC25A1 | Thermo Fisher Scientific | Hs01105608_g1 |
| SLC25A10 | Thermo Fisher Scientific | Hs00201730_m1 |
| SLC25A11 | Thermo Fisher Scientific | Hs01087948_g1 |
| MDH1 | Thermo Fisher Scientific | Hs00936497_g1 |
| HPRT1 | Thermo Fisher Scientific | Hs02800695_m1 |
| **Chemicals, Peptides, and Recombinant Proteins** | | |
| 7-AAD | Sigma-Aldrich (Merck) | Cat# A9400 |
| CHIR99021 | R&D Systems | Cat# 4423/10 |
| Activin A | R&D Systems | Cat# 338-AC |
| Hyclone Penicillin-Streptomycin Solution | Thermo Fisher Scientific | Cat# SV30010 |
| KnockOut Serum Replacement | Thermo Fisher Scientific | Cat# 10828010 |
| ROCK-Inhibitor (Y-27632) | Sigma-Aldrich (Merck) | Cat# SCM075 |
| CNASB (4-chloro-3-3-nitrophenyl-amino-sulfonylbenzoic acid) | Sigma-Aldrich (Merck) | Cat# SML0068 |
| Tetramethylrhodamine, Ethyl Ester, Perchlorate (TMRE) | Thermo Fisher Scientific | Cat# T669 |
| Myxothiazol | Sigma-Aldrich (Merck) | Cat# T5580 |
| Hydrogen peroxide solution, 30% (w/w) | Sigma-Aldrich (Merck) | Cat# H1009 |
| Rotenone | Sigma-Aldrich (Merck) | Cat# R8875 |
| Antimycin A | Sigma-Aldrich (Merck) | Cat# A8674 |
| StemPro Accutase Cell Dissociation Reagent | Thermo Fisher Scientific | Cat# A1110501 |
| Hyclone Penicillin-Streptomycin Solution | Thermo Fisher Scientific | Cat# SV30010 |
| GlutaMAX Supplement | Thermo Fisher Scientific | Cat# 35050038 |
| Recombinant Human SCF | Peprotech | Cat# 300-07 |
| Recombinant Human IL6 | Peprotech | Cat# 200-06 |
| Recombinant Human BMP4 | Peprotech | Cat# AF-120-05ET |
| Recombinant Human VEGF 165 | Peprotech | Cat# AF-100-20 |
| Recombinant Human GM-CSF | Peprotech | Cat# 300-03 |
| Recombinant Human IL-3 | Peprotech | Cat# 200-03 |
| Recombinant Human FGF-basic | Peprotech | Cat# 100-18B |
| Recombinant Human Flt3-L | Peprotech | Cat# 300-19 |
| Recombinant Human IGF-I | Peprotech | Cat# 100-11 |
| Recombinant Human IL-11 | Peprotech | Cat# AF-200-11 |
| Retacrit 10 000 IU/1 mL solution (EPO) | Hospira UK Ltd |  |
| DMEM-F12 | Thermo Fisher Scientific | Cat# 31330 |
| RPMI 1640 Medium | Thermo Fisher Scientific | Cat# 11875093 |
| StemPro-34 SFM (1X) | Thermo Fisher Scientific | Cat# 10639011 |
| Ascorbic Acid | Sigma-Aldrich (Merck) | Cat# A4544 |
| Glutamax Supplement | Thermo Fisher Scientific | Cat# 35050061 |
| MEM Non-Essential Amino Acids Solution | Thermo Fisher Scientific | Cat# 11140050 |
| 2-Mercaptoethanol (50 mM) | Thermo Fisher Scientific | Cat# 31350010 |
| N2 (100x) | Thermo Fisher Scientific | Cat# 17502048 |
| B27 minus VitA (50x) | Thermo Fisher Scientific | Cat# 12587010 |
| holo-Transferrin human | Sigma-Aldrich (Merck) | Cat# T0665 |
| Losartan potassium | Tocris (R&D Systems) | Cat# 3798 |
| Angiotensin II human | Sigma-Aldrich (Merck) | Cat# A9525 |
| Recombinant Human Sonic Hedgehog/Shh | R&D Systems | Cat# 1314-SH-025/CF |
| FBS | Thermo Fisher Scientific | Cat# SV30160 |
| rhLaminin-521 | Thermo Fisher Scientific | Cat# A29249 |
| Matrigel Matrix | Corning | Cat# 354230 |
| TrypLE Express | Thermo Fisher Scientific | Cat# 12604-013 |
| HyClone Phosphate Buffered Saline (PBS) | Thermo Fisher Scientific | Cat# 10462372 |
| CryoStor CS10 | STEMCELL TECHNOLOGIES | Cat# 07930 |
| Nuclei Buffer (20X) | 10x Genomics | Cat# 2000153 |
| Digitonin | Thermo Fisher Scientific | Cat# BN2006 |
| Trizma Hydrochloride Solution, pH 7.4 | Sigma-Aldrich (Merck) | Cat# T2194 |
| Sodium Chloride Solution, 5 M | Sigma-Aldrich (Merck) | Cat# 59222C |
| Magnesium Chloride Solution, 1M | Sigma-Aldrich (Merck) | Cat# M1028 |
| IGEPAL CA-630 | Sigma-Aldrich (Merck) | Cat# i8896 |
| MACS BSA Stock Solution | Miltenyi Biotec | Cat# 130-091-376 |
| Tween 20 | Bio-Rad | Cat# 1662404 |
| 1X Phosphate-Buffered Saline, pH 7.4 | Corning | Cat# 21-040-CV |
| **Critical Commercial Assays** | | |
| CD34 MicroBead Kit, human | Miltenyi Biotec | Cat# 130-046-703 |
| CellTrace Violet Cell Proliferation Kit | Thermo Fisher Scientific | Cat# C34571 |
| Chromium Next GEM Single Cell Multiome ATAC + Gene Expression Reagent kit | 10x Genomics | Cat# 1000283 |
| Click-iT EdU Alexa Fluor 647 Flow Cytometry Assay Kit | Thermo Fisher Scientific | Cat# C10424 |
| CellROX Green Flow Cytometry Assay Kit | Thermo Fisher Scientific | Cat# C10492 |
| BODIPY 581/591 C11 Lipid Peroxidation Sensor | Thermo Fisher Scientific | Cat# D3861 |
| ThiolTracker Violet | Thermo Fisher Scientific | Cat# T10095 |
| BD Pharmingen Apoptosis detection kit | BD Biosciences | Cat# 559763 |
| MitoPlate S-1 | Biolog | Cat# 14105 |
| \| RNeasy Mini Kit \|  \|  \| \| --- \| --- \| --- \| | QIAGEN | Cat# 74104 |
| TaqMan Gene Expression Master Mix | Thermo Fisher Scientific | Cat# 4369542 |
| GeneJET Plasmid Miniprep Kit | Thermo Fisher Scientific | Cat# K0502 |
| NucleoBond Xtra Midi prep kit | Macherey-Nagel | Cat# 740410.50 |
| True-Nuclear Transcription Factor Buffer Set | BioLegend | Cat# 424401 |
| LIVE/DEAD Fixable Red Dead Cell Stain Kit | Thermo Fisher Scientific | Cat# L34971 |
| **Flow Cytometry antibodies** | | |
| CD144 (VE-Cadherin), Clone 55-7H1 | BD Biosciences | Cat# 561566, RRID:AB_10715835 |
| CD43, Clone 1G10 | BD Biosciences | Cat# 655407 |
| CD34, Clone 561 | BioLegend | Cat# 343604, RRID:AB_1732005 |
| CD90, Clone 5E10 | BD Biosciences | Cat# 562685, RRID:AB_2744468 |
| GPA, Clone HIR2 (GA-R2) | Thermo Fisher Scientific | Cat# 48-9987-42, RRID:AB_2574141 |
| CD45, Clone HI30 | BioLegend | Cat# 304024, RRID:AB_493761 |
| CD73, Clone AD2 | BD Biosciences | Cat# 550257, RRID:AB_393561 |
| CD14, Clone 63D3 | BioLegend | Cat# 367104, RRID:AB_2565888 |
| CD11b, Clone ICRF44 | BioLegend | Cat# 301310, RRID:AB_314162 |
| CD33, Clone WM-53 (WM53) | Thermo Fisher Scientific | Cat# 25-0338-42, RRID:AB_1907380 |
| CD184 (CXCR4), Clone 12G5 | BD Biosciences | Cat# 555976, RRID:AB_398616 |
| CoraLite Plus 488-conjugated anti-NRF2, NFE2L2 Polyclonal antibody | Proteintech | Cat# CL488-16396-100UL |
| Anti-NFE2 Polyclonal antibody (Rabbit) | Proteintech | Cat# 11089-1-AP |
| Anti-BACH1 (Rabbit) | Sigma-Aldrich (Merck) | Cat# B1310-200UL |
| Donkey anti-Rabbit IgG (H+L) Secondary Antibody | Thermo Fisher Scientific | Cat# R37118 |
| **Deposited Data** | | |
| The Multiome ATAC + RNA sequencing data presented in this paper is available in the GEO database. |  | accession number GSE270141 |
| **Plasmids** | | |
| pLV hU6-sgRNA hUbC-dCas9-KRAB-T2a-GFP (From Charles Gersbach PMID 26501517) | Addgene | Cat #71237  RRID:Addgene_71237 |
| **Experimental Models: Cell Lines** | | |
| iPSC-CB1RB9 cell line, human (RB9-CBiPS2) | Woods et al, STEM CELLS (2011) | PMID: 21544903 |
| Mouse Embryonic Fibroblasts | Merck-Millipore | Cat# PMEF-NL |
| K562 cell line | ATCC | Cat# CCL-243 |
| **Software and Algorithms** | | |
| FlowJo | BD Life Sciences | https://www.flowjo.com/solutions/flowjo/ downloads |
| GraphPad Prism 10 | GraphPad | http://www.graphpad.com/support/faqid/%201952 |
| FACSDiva v8.0.1 | BD Bioscience | https://www.bdbiosciences.com/en-us/instruments/research-instruments/research-software/flow-cytometry-acquisition/facsdiva-software |
| Cytoscape 3.10.2 | Shannon et al, Genome Res (2003) | PMID: 14597658 |
| Adobe Illustrator 2024 | Adobe Systems Inc | https://www.adobe.com/cn/products/cs6/illustrator.html |
| RStudio (v1.2.1578) |  | https://rstudio.com/about/ |
| Seurat package v3.1.0 | Butler et al, Nat Biotech (2018); Stuart et al, Cell (2019) | PMID: 29608179  PMID: 31178118 |
| dittoSeq package v3.18 | Bunis et al, Bioinformatics (2020) | PMID: 33313640 |
| fgsea package | Korotkevich et al, bioRxiv | https://doi.org/10.1101/060012 |
| STACAS package | Andreatta et al, Bioinformatics (2021); Andreatta et al, Nat Comm (2024) | PMID: 32845323  PMID: 38287014 |
| ToppCluster | Kaimal et al, Nucleic Acids Res (2010) | PMID: 20484371 |
| MACS2 package | Zhang et al, Genome Biology (2008) | PMID: 18798982 |
| Signac package | Stuart et al, Nat Met (2021) | PMID: 34725479 |
| DoRothEA regulons | Garcia-Alonso et al, Genome Res. (2019) | PMID: 31340985 |
